# Open Science Meets Stem Cells: A New Drug Discovery Approach for Neurodegenerative Disorders

**DOI:** 10.3389/fnins.2018.00047

**Published:** 2018-02-06

**Authors:** Chanshuai Han, Mathilde Chaineau, Carol X.-Q. Chen, Lenore K. Beitel, Thomas M. Durcan

**Affiliations:** Montreal Neurological Institute and Hospital, McGill University, Montreal, QC, Canada

**Keywords:** neurodegenerative diseases, drug discovery, induced pluripotent stem cells, Open Science, iPSC-derived neurons, Alzheimer's disease, Parkinson's disease, amyotrophic lateral sclerosis

## Abstract

Neurodegenerative diseases are a challenge for drug discovery, as the biological mechanisms are complex and poorly understood, with a paucity of models that faithfully recapitulate these disorders. Recent advances in stem cell technology have provided a paradigm shift, providing researchers with tools to generate human induced pluripotent stem cells (iPSCs) from patient cells. With the potential to generate any human cell type, we can now generate human neurons and develop “first-of-their-kind” disease-relevant assays for small molecule screening. Now that the tools are in place, it is imperative that we accelerate discoveries from the bench to the clinic. Using traditional closed-door research systems raises barriers to discovery, by restricting access to cells, data and other research findings. Thus, a new strategy is required, and the Montreal Neurological Institute (MNI) and its partners are piloting an “Open Science” model. One signature initiative will be that the MNI biorepository will curate and disseminate patient samples in a more accessible manner through open transfer agreements. This feeds into the MNI open drug discovery platform, focused on developing industry-standard assays with iPSC-derived neurons. All cell lines, reagents and assay findings developed in this open fashion will be made available to academia and industry. By removing the obstacles many universities and companies face in distributing patient samples and assay results, our goal is to accelerate translational medical research and the development of new therapies for devastating neurodegenerative disorders.

## Introduction

Neurodegenerative disorders, such as Alzheimer's disease (AD), Parkinson's disease (PD), and amyotrophic lateral sclerosis (ALS), are incurable and debilitating conditions characterized by progressive degeneration of specific neurons within the brains of affected individuals. According to the World Alzheimer Report 2016, there are 46.8 million people living with dementia in the world (Prince et al., 2016). The total estimated worldwide cost of dementia in 2015 was $818 billion US, and is forecast to rise to over a trillion dollars by 2018. The Parkinson's Disease Foundation estimates seven to 10 million people worldwide are living with PD. Medication costs an average of $2,500 per year per patient (Parkinson's Foundation, 2017) and associated costs markedly increase over time (Martinez-Martín et al., 2015; Bovolenta et al., 2017); therapeutic surgery can cost up to $100,000 (Parkinson's Foundation, 2017). ALS Worldwide reports more than 500,000 people around the world currently suffer from ALS, with an average life expectancy of about 2 to 5 years from the time of diagnosis (Naqvi, 2017). Moreover, the average cost of ALS to a family over the course of the disease can be $150,000 to $250,000 (Arthur et al., 2016). Since the incidence of neurodegenerative conditions increases significantly with age, and world populations are rapidly ageing, the number of people with dementia or PD is expected to reach up respectively to 131.5 million and 8.7 million by 2040 (Kowal et al., 2013), while the number of people with ALS is estimated to increase to 377,000 (Arthur et al., 2016). Thus, for countries throughout the world, neurodegenerative diseases have become an enormous economic burden that is projected to grow significantly over the next few decades in the absence of any new therapeutic interventions.

Despite massive investments in drug discovery and growing numbers of molecules in development, there are still no cures or disease-modifying therapies for neurodegenerative diseases. Currently available therapies only help manage symptoms of these disorders, and none identified to date can halt or prevent progression of these disorders. Only four drugs are approved and currently used in symptomatic treatment for AD: acetylcholinesterase inhibitors, including donepezil (1997), rivastigmine (2000), and galantamine (2001), to ameliorate the clinical manifestations of AD by enhancing cholinergic neurotransmission in relevant parts of the brain (Birks et al., 2000; Olin and Schneider, 2002; Cacabelos, 2007); and memantine (2003), a N-methyl-D-aspartate receptor antagonist for improving AD behavioral symptoms (van Marum, 2009). For PD, L-dihydroxyphenylalanine (L-DOPA), combined with peripheral inhibitors of L-amino acid aromatic decarboxylase (carbidopa and benserazide) is still the gold-standard of care (LeWitt, 2015) but unfortunately, the beneficial effects of L-DOPA are not permanent and motor fluctuations and dyskinesia occur after a few years of treatment (Guridi et al., 2012). Also, none of the current anti-parkinsonian agents, including L-DOPA, has shown convincing activity as a disease modifier. Rilutek (also known as riluzole), was the first medication that the FDA approved specifically for the systemic treatment of ALS. Although it helps slow down the progression of ALS/motor neuron disease and prolongs survival, it does not cure ALS nor reverse nerve damage or muscle weakness (Petrov et al., 2017). Edaravone, a free radical scavenger approved by the FDA in May 2017, is only effective in specific well-defined types of early stage ALS and there is no evidence showing it can prolong survival (Hardiman and van den Berg, 2017).

## The classical drug discovery pipeline

Hence, only a limited number of drugs are currently available for treatment of neurodegenerative disorders, and despite increased investment in R&D for the past seven decades, the number of new drugs brought to market by pharmaceutical companies has not increased accordingly (Munos, 2009). The classical drug discovery pipeline comprises different stages, with the first step using a target or phenotype-driven drug screen to identify one or more small molecules. Candidate molecules with the largest effect are next directed into medicinal chemistry programs, to modify their structures and enhance specificity, efficacy and stability. One or two lead compounds are tested in animals to determine the molecule's toxicity, and optimal dose and delivery route. Following success in cell and animal models, the lead molecule is brought forward to a phase I trial to test the safety of the molecule in humans, before being tested for efficacy in an increased number of patients in phase II and III clinical trials. After completion of phase III, the candidate drug must be approved by relevant regulatory agencies such as the Food and Drug Administration (FDA) in the US, the Health Products and Food Branch in Canada, or the European Medicines Agency in European Union, before being released to the market. According to the Pharmaceutical Research and Manufacturers of America, developing a new medicine costs $2.6 billion on average from drug discovery to FDA approval. Drug discovery and development is inherently risky, with recent figures indicating that less than 11% of new pharmaceutical agents that entered clinical development reached the marketplace across all therapeutic areas (DiMasi et al., 2003). Drugs for the central nervous system, including neurodegenerative diseases, that entered clinical development, have a considerably lower probability of reaching the marketplace (7%) than the industry average across other therapeutic areas (15%), and require a longer time for development and regulatory approval (average of 12.6 years) compared with most other diseases (e.g., 6.3 years for cardiovascular and 7.5 years for gastrointestinal indications) (Kola and Landis, 2004; Pangalos et al., 2007). In AD, for example, the cost of developing a disease-modifying therapy, including the cost of failures, is currently estimated at $5.7 billion (Scott et al., 2014). However, over 100 compounds tested as potential therapies were either abandoned in development or failed in clinical trials, e.g., a negative Phase III trial of the once-promising AD therapy solanezumab (Doody et al., 2014), and a halted late-stage trial on the drug verubecestat for AD (Hawkes, 2017).

## Challenges in drug discovery

Developing new therapies requires a deep understanding of the genes and targets that drive neuronal death. While our understanding of these disorders has advanced significantly, the complexity of the brain and a lack of access to human tissue has hindered progress. Consequently, current models, including cell and animal models, may not predict whether a drug candidate is likely to modify disease progression or improve patient behavior. Another barrier in current drug development is the lack of transparency in communicating and sharing of data and reagents. Most studies, including clinical trials, keep their data and biospecimens behind restrictive firewalls and material transfer agreements (MTAs) and only publish positive results, leaving large amounts of negative, but potentially meaningful data lying dormant. Hence, it is essential to improve the current drug development process for neurodegenerative diseases, to efficiently share clinical samples and research data, and to find strategies that lower the cost, time, and risk in delivering new therapies.

## Classical cellular and animal models

While we are beginning to understand the cellular pathways involved in neurodegenerative diseases, many experimental studies and drug trials have been based on results from laboratory-grown cell lines and experimental animal models. Attempts at translating “cures” from mice to humans have been largely unsuccessful for neurodegenerative diseases, due to fundamental species-specific differences. The relatively short lifespan of rodents may not allow for development of clear-cut neurodegenerative phenotypes, while acute models may not accurately represent the mechanisms underlying chronic neurodegeneration. Although results from animal models may predict drug efficacy for symptomatic treatment, they are less helpful for identifying drugs that potentially act as disease modifiers. Thus, many clinical trials arising from preliminary work in animal models have failed (Becker, 2007; Mehta et al., 2017). In term of cellular models, most groups use immortalized fibroblasts, nervous system tumors, or immortalized neuronal progenitor cell lines for *in vitro* assays to identify potent agents with desired selectivity profiles. Although these cell lines grow readily at a relatively low cost, they generally cannot fully represent critical features of endogenous neural cells, and often fail to reflect relevant disease pathways. One strategy to overcome this hurdle would be drug screening in the most relevant cell-types (e.g., cholinergic basal forebrain neurons for AD, dopaminergic neurons for PD, and motor neurons for ALS) obtained from patients afflicted with the disease. In cancer, access to such patient material (via tumor resections or biopsies) has led to a revolution in new therapies, with many patients now bypassing toxic chemotherapy regimens for newer targeted personalized therapies, based on their tumor biology (Goodspeed et al., 2016). Unfortunately, access to relevant patient-derived cells has been a major hurdle in neurodegenerative diseases, as biopsies or resections to obtain neurons are rarely, if ever, carried out in patients afflicted with these diseases.

## Induced pluripotent stem cells as a new drug discovery model for neurodegenerative diseases

**Figure 1:**
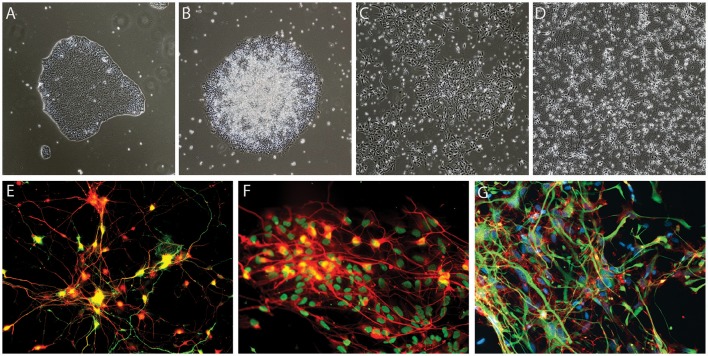
Examples of iPSC-derived cells. **(A–E)** Generation of dopaminergic neurons from human iPSCs based on the protocol developed by Kriks et al. (2011). Brightfield images of iPSC neural differentiation, **(A)** iPSCs, **(B)** neural rosettes, **(C)** dopaminergic neural precursor cells, **(D)** dopaminergic neurons, and **(E)** tyrosine hydroxylase (TH)-expressing dopaminergic neurons derived from iPSCs, TH in green and pan-neuronal marker beta-III tubulin in red **(F)** example of motoneurons generated based on the protocol of Du et al. (2015), HB9 in green and pan-neuronal marker beta-III tubulin in red **(G)** iPSCs-derived glial fibrillary acidic protein (GFAP)-expressing astrocytes generated using Krencik and Zhang's protocol (Krencik and Zhang, 2011), GFAP in green and CD44 in red.

The discovery of the Yamanaka factors more than a decade ago (Takahashi and Yamanaka, 2006), has led to a paradigm shift in stem cell biology, providing the tools to efficiently generate human induced pluripotent stem cells (iPSCs) using skin (Takahashi et al., 2007), blood (Loh et al., 2009) or urine-derived cells (Zhou et al., 2011). Under the appropriate conditions, iPSCs can be differentiated into any cell type, including neurons. This technology has opened a new avenue for research, allowing scientists access to human neurons and other cell types involved in neurodegenerative diseases, such as astrocytes, microglial cells and oligodendrocytes, in an unlimited manner (Figure 1). Disease-related phenotypes in patient iPSC-derived neurons are undoubtedly helpful for understanding disease mechanisms and pursuing potential treatments, and to bridge the gap between current pre-clinical research and clinical testing by giving us a better predictive value than current animal and cellular models.

The development of iPSC technology makes it possible to acquire disease-specific cell lines from patients carrying familial mutations and these cell types show exciting promise for the elucidation of neurodegenerative disease etiology. A few clinical trials have been initiated based on results obtained by using iPSC technology. Bright and colleagues generated iPSCs from patients with sporadic or presenilin-1-mutant AD. By comparing the cortical neurons derived from these AD patients and age-matched controls, they discovered the AD-derived neurons secreted a specific form of Tau and they developed BMS-986168 as a specific antibody for the Tau fragments (Bright et al., 2015). In 2017, BMS-986168, licensed by Biogen, entered Phase II clinical trials for AD, and progressive supranuclear palsy. In another study, iPSC-derived motor neurons from ALS patients were found to be hyperexcitable compared to controls, and Retigabine, an approved drug for epilepsy, could rescue this hyperexcitability phenotype in motor neurons derived from patients with different ALS-associated mutations (Wainger et al., 2014). Presently, Retigabine is under a placebo-control Phase II clinical trial with 192 ALS patients in collaboration with GlaxoSmithKline. Moreover, iPSC-derived neurons also create opportunities to study sporadic forms of neurodegenerative diseases, which are the vast majority, and in which the causes remain largely unknown. There is a growing number of iPSC lines derived from patients with sporadic AD, PD, or ALS (Qian et al., 2017; Zhang et al., 2017). These cell lines, which carry different genetic risk variants, will help us obtain better insights into the pathogenesis of sporadic disorders, and will be useful *in vitro* cellular models for drug discovery. For example, motor and cortical neurons differentiated from sporadic ALS patients show *de novo* TDP-43 aggregation, which is one of the observed pathologies in postmortem tissue from ALS patients (Burkhardt et al., 2013). Using the TDP-43 aggregation phenotype as readout in a high-content chemical screen in lower and upper motor neuron-like cells, the authors also identified previously approved drugs with known targets that could modulate TDP-43 aggregation. Moreover, iPSC-derived disease models are starting to be used for drug discovery for other neurological diseases including spinal muscular atrophy (Ando et al., 2017), multiple sclerosis (Miquel-Serra et al., 2017) and autism spectrum disorders (Mokhtari and Lachman, 2016). With the technology to reprogram and generate selected types of functional neurons, iPSCs are also widely considered to have good potential for cell replacement therapy in neurodegenerative diseases. Intriguingly, neural precursor cells differentiated from reprogrammed iPSCs were reported to migrate into various brain regions upon transplantation, to differentiate into glia and neurons, including dopaminergic neurons, and to improve behavior in both rodent and primate models of PD (Wernig et al., 2008; Kikuchi et al., 2017). However, it is important to note that key questions related to safety and efficacy of such therapy still need to be addressed before clinical trials of stem cell-based transplantation for PD (Barker et al., 2017).

With the advent of iPSCs, a burgeoning pharmaceutical and biotechnology field has emerged (Passier et al., 2016). In early years, several start-ups were founded, focused on using iPSCs for deriving human cells for safety studies, small molecule screens and *in vitro* disease modeling. Several companies proved successful at deriving neurons and other cell types to sell to pharmaceutical industries and to other users for drug toxicity testing or for further research. Other start-ups focused on developing new drug discovery platforms for neurodegenerative diseases using iPSC-derived neurons. More companies are now starting to take advantage of iPSCs to generate new clinical products, using iPSC-based disease models to bring new therapies into clinical trials. IPSC technology has advanced significantly in the last 5 years, reducing both the time and cost involved. In parallel, new genes and pathways have been identified that can be harnessed to develop disease-relevant assays. We foresee that using iPSC technology to probe disease mechanisms and screen for new drugs will effectively usher in a new era of therapeutics and personalized medicine for devastating neurodegenerative diseases.

However, under the current model of MTAs and legal agreements, accessing iPSCs can be cumbersome, with complex legal agreements required before researchers can access cell lines. Moreover, researchers are often heavily restricted in their use of these lines, and even in how they can disseminate their findings. All these obstacles increase the time and efforts required to go from the bench to clinic, adding to the already long drug development pipeline. Thus, we believe that removing these restrictions and streamlining the process in a more “open” manner, and making iPSCs and all data generated from these lines openly available to the research community, will help to accelerate the drug discovery process.

## Open science, a new path for research and development

The goal of Open Science is to accelerate research and discovery by encouraging collaborations and partnerships. The term “Open Science” embraces different levels of openness, from “open data” which implies sharing results with the scientific community, to an “open access” model in which every step of the research process should be transparent to the community. Such a model would mandate that results, publications, reagents, compounds and even clinical trials results are accessible to the public and all groups without restriction. Using iPSC technology to study neurodegenerative diseases will lead to an increased number of biological samples collected and amount of data generated. Adopting an Open Science policy is one strategy to build an efficient infrastructure to support the exploration, integration and utilization of existing data and biological samples resources to accelerate drug discovery in neurodegenerative diseases. Among the most famous Open Science initiatives are the Human Genome Project and the Allen Institute. A formal agreement to encourage free distribution of research data, technology and resources created by the Human Genome Project has already had a major input on research across the life sciences. Especially, it brings important genetic clues to understanding diseases in terms of human biology and pathology, which is “starting to have profound impact on biomedical research and promises to revolutionize the wider spectrum of biological research and medical medicine” (Kelavkar, 2001). The Allen Institute, founded in 2003, quickly became a powerful resource for brain scientists worldwide by freely sharing gene-expression maps for human and mouse brains (Siegle et al., 2017).

The Open Science era is also expected to be of great benefit to drug development by increasing partnerships between academia and pharmaceutical companies, and eliminating barriers between the different stages of drug development. A successful example is the “Pathogen Box,” which is an open-access collection comprised of 400 compounds with demonstrated biological activity against specific pathogenic organisms that cause tropical and neglected diseases. Upon request, researchers around the world will receive a Pathogen Box of molecules to help catalyze neglected disease drug discovery. In return, researchers are asked to share any data generated in the public domain, creating an open and collaborative forum for neglected diseases drug research (Duffy et al., 2017). One of the successes from “Pathogen Box” has been published with the identification of *Candida albicans* biofilm inhibitor (Vila and Lopez-Ribot, 2017). Another precedent is the sharing of the chemical probe JQ1. JQ1 is a small molecule targeting bromodomain proteins that regulate protein-histone association and chromatin remodeling. After the discovery of JQ1's effect on specific cancer cells (Filippakopoulos et al., 2010), the researcher released all the information on JQ1 and distributed samples of JQ1 to academic and industrial laboratories worldwide. This open-access manner considerably accelerated drug discovery for this class of compound, not only in the field of cancer, but also for other diseases, including neurodegenerative diseases (Scott, 2016). According to the data from ClinicalTrials.gov, there are 21 Phase I clinical trials, 2 Phase I/II trials, and 1 Phase III trial for bromodomain inhibitors (Xu and Vakoc, 2017). There are more and more initiatives sharing well-characterized preclinical compounds with the whole research community. Boehringer Ingelheim recently launched a platform opnME portal to share nearly 20 high-quality chemical probes, without intellectual property restrictions (Mullard, 2017). The Structural Genomic Consortium (SGC) is another outstanding example of an organization involved in advancing the Open Science model. SGC, founded in 2004, represents a worldwide partnership between universities and pharmaceutical companies. The key to making this model work is the combination of different principles, including a full commitment by scientists in exchange for predictable funding, as long as they meet their milestones, and a requirement for data sharing and increasing reproducibility (e.g., by using electronic lab notebooks) (Edwards, 2016a). Currently, SGC scientists from six universities are collaborating with scientists from nine large pharmaceutical companies to test the effects of compounds and chemical probes in primary human cells from patients with different diseases, such as cancer and inflammatory and auto-immune diseases (Edwards, 2016b). Recently, SGC started a collaboration with the MNI, as part of the Neuro Open Science initiative, to screen their compounds on iPSC-derived cells from patients with PD and ALS.

As discussed above, the Open Science model can help the pharmaceutical industry and academics to work together to advance the discovery and development of medicines. However, intellectual property is a key concern in this model. In the pharmaceutical industry, with multibillion dollar investments in molecules that can easily be recreated by competitors, investors require proof of protection of their assets. It is therefore natural to assume that open access may jeopardize this, as it is a widely held belief that an exclusivity period is required for an organization to profit from a new drug. However, in the case of open access to JQ1, there has been a clear increase in research activity around the bromodomain proteins, leading to more than 100 filed patents. These patents are not for JQ1 itself, but for other molecules that target bromodomains; the development of many of these was guided by the use of JQ1 as a research tool (Arshad et al., 2016). With a wider, multidisciplinary research community contributing to higher impact research into the molecule itself, the initial free availability of the JQ1 molecule led to increased downstream patenting. This offers evidence that open access is a commercially viable model for drug discovery with the potential to lead to improved commercial gain for drug developers in the long run. By allowing the initial stages of drug development to be carried out in an Open Science model, many researchers can benefit from the availability of information regarding drug candidates during a time of high risk and attrition. The Open Science environment would allow this high risk to be distributed among different stakeholders, all the while facilitating downstream patenting, allowing inventors to benefit from their inventions at a later and more commercially viable stage of drug translation. This could in fact lead to greater profit for an industry that has been suffering from declining reimbursement in the past few decades.

## Open science meets iPSCs

**Figure 2:**
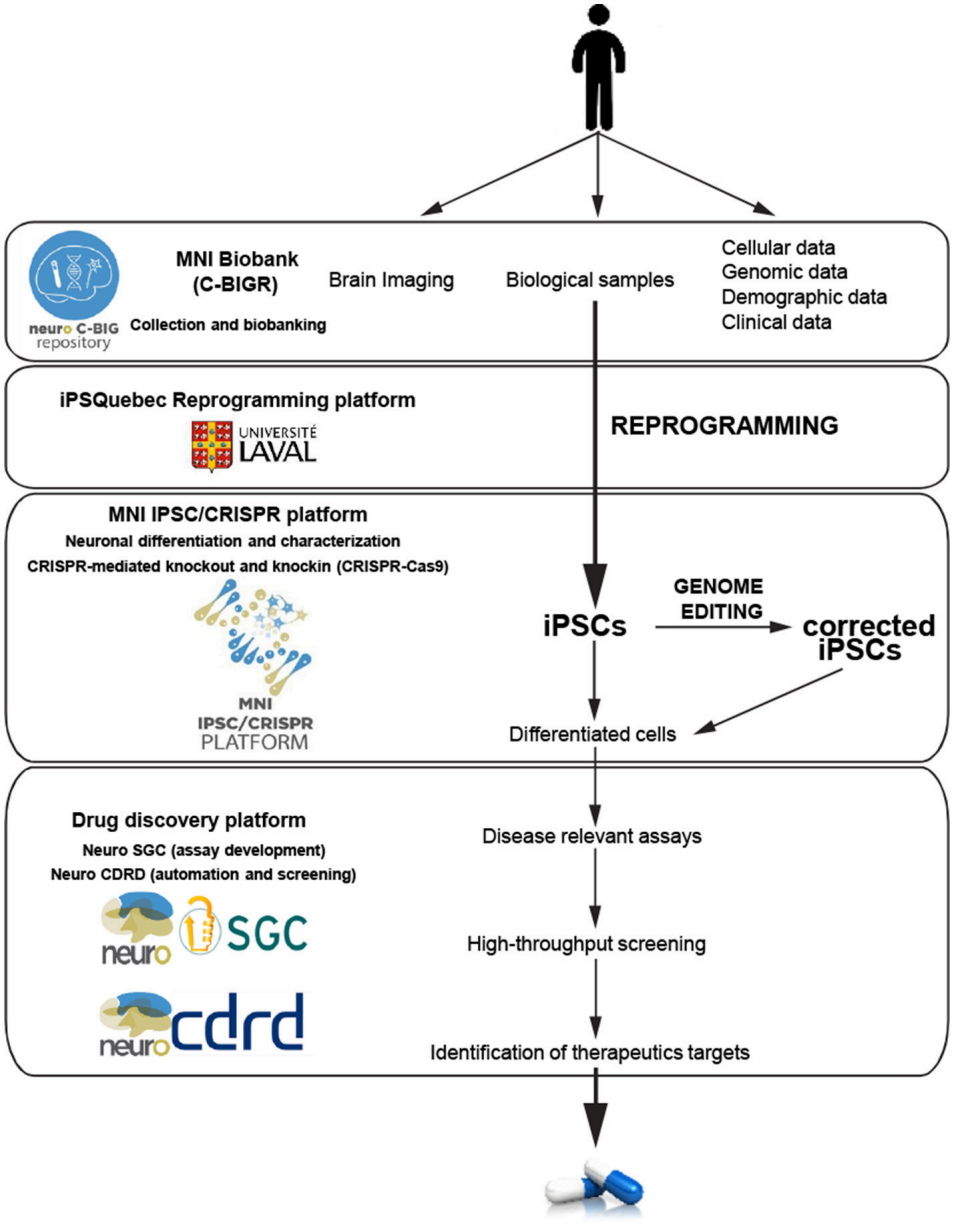
Schematic of the hiPSC neurodegenerative disease modeling for drug discovery at the MNI. Data and biological samples from patients with neurodegenerative diseases are collected and banked by the C-BIGR. hiPSC derived from somatic cells from patients are characterized and isogenic controls are created by the iPSC-CRISPR platform. IPSC are differentiated into specific cells including different types of neurons, astrocytes and glial cells. Relevant differentiated cells are then used by the drug discovery platform to develop disease-relevant assays to screen for therapeutic targets.

Seeking to accelerate the generation of knowledge and to develop novel effective treatments for brain disorders, the MNI is adopting an institutional Open Science policy that includes five aspects: open access, open data, open intellectual property, open sharing of biological samples and other resources, and open commercialization (Poupon et al., 2017). As the first academic research institution to develop an Open Science framework, a robust cyberinfrastructure platform plays a critical role in allowing sharing of data and materials. The MNI implemented its own cyberinfrastructure, using the LORIS and C-Brain platforms developed at the MNI by Dr. Evans, and have made a vast amount and variety of data easily accessible (Das et al., 2016). Beyond cyberinfrastructure, two other key components to stimulate drug discovery are the MNI Open Clinical Biological Imaging and Genetic Repository (C-BIGR), and the Open Drug Discovery Platform (ODDP). The C-BIGR is a freely-shared source of information linked to biomedical specimens, based on the strategy that deep phenotypic information about each patient will be obtained, as well as a variety of biological samples. Under the supervision of the Research Ethics Board, C-BIGR created an information and consent form for patients, outlining how their biological material and data will be used and stored; and also developed an encrypted system to protect all related personally identifiable information. Within this infrastructure, C-BIGR is designed to curate brain imaging, clinical, demographic, genetic (DNA), and cell data, along with biological samples from patients with neurological disorders, all of which will be made openly available to users upon request. The Open Drug Discovery Platform includes the MNI iPSC/CRISPR platform, Neuro-SGC (assay development), and Neuro-CDRD (Center for Drug Research and Development) (automation and screening). These combined platforms will use iPSCs derived from C-BIGR samples to create disease-relevant assays that should facilitate accurate therapeutic target identification, and bring new drugs more rapidly to market (Figure 2).

Combining iPSC technology and Open Science infrastructure will be advantageous for accelerating and disseminating developments in disease-modifying therapies. First, through collaborations between C-BIGR and the iPSC/CRISPR platform, we have direct access to neurons generated from patient-derived iPSCs to study mechanisms of neurodegenerative diseases. These studies will greatly enhance our knowledge and provide valuable information on potential drug targets. Secondly, with an “Open Access” policy, all research results and observations will be published on MNI Open Research, a science publishing platform (https://mniopenresearch.org/), in a nearly-immediate and no-restriction way, including negative results. This policy will provide support for research integrity, reproducibility and transparency, which are the foundations for success of translational medical research. Sharing data openly can also bring about the opportunity to explore existing data in a worldwide collaborative efficient manner, which should directly accelerate neurodegenerative disease drug discovery.

Developments in iPSC technology and other rapid advances in cellular and molecular neurobiology, wide collaboration between industry/pharma, clinicians and academic researchers, and commitment to an Open Science philosophy will be the future driving forces to accelerate development of disease-modifying therapies for neurodegenerative diseases, and to spark further discovery and development, including commercialization. By working together in this open manner, we are hopeful this innovative approach will accelerate the development of new treatments for the millions of people with neurodegenerative diseases.

## Author contributions

CH, MC, CC, LB, and TD participated in conception and design. CH, MC, and CC drafted the manuscript. CH, MC, LB, and TD revised the manuscript for important intellectual content.

### Conflict of interest statement

The authors declare that the research was conducted in the absence of any commercial or financial relationships that could be construed as a potential conflict of interest.
